# Transcriptomic and Clinical Profiling Reveals *LGALS3* as a Prognostic Oncogene in Pancreatic Cancer

**DOI:** 10.3390/genes16101170

**Published:** 2025-10-03

**Authors:** Grazia Scuderi, Sanja Mijatovic, Danijela Maksimovic-Ivanic, Michelino Di Rosa, José Francisco Muñoz-Valle, Alexis Missael Vizcaíno-Quirarte, Gian Marco Leone, Katia Mangano, Paolo Fagone, Ferdinando Nicoletti

**Affiliations:** 1 Department of Biomedical and Biotechnological Sciences, University of Catania, 95123 Catania, Italy; graziascuderi@hotmail.it (G.S.); mdirosa@unict.it (M.D.R.); g.marco-94@outlook.it (G.M.L.); kmangano@unict.it (K.M.); ferdinic@unict.it (F.N.); 2 Department of Immunology, Institute for Biological Research “Sinisa Stankovic”, Belgrade University, 11060 Belgrade, Serbia; sanjamama@yahoo.com (S.M.); nelamax@yahoo.com (D.M.-I.); 3 Department of Molecular Biology and Genomics, University Center of Health Sciences, University of Guadalajara, Guadalajara 44340, Jalisco, Mexico; biologiamolecular@hotmail.com (J.F.M.-V.); alexis.vizcaino@cucs.udg.mx (A.M.V.-Q.); 4 Department of Basic Psychology, University Center of Health Sciences, University of Guadalajara, Guadalajara 44340, Jalisco, Mexico

**Keywords:** galectins, *LGALS3*, transcriptomics, prognostic biomarker

## Abstract

Background/Objectives: Galectin-3 (Gal-3), encoded by *LGALS3*, is a β-galactoside-binding lectin involved in diverse tumor-associated processes, including immune modulation, cell cycle regulation, and stress adaptation. Despite its known roles in cancer biology, the full extent of its molecular functions and prognostic relevance across tumor types remains incompletely understood. This study aimed to systematically investigate the transcriptomic impact of *LGALS3* deletion and assess its clinical significance in cancer. Methods: We analyzed CRISPR-Cas9 knockout transcriptomic data from the SigCom LINCS database to characterize the consensus gene signature associated with *LGALS3* loss using functional enrichment analyses. Pan-cancer survival analyses were conducted using TIMER2.0. Differential Gal-3 protein levels in ductal adenocarcinoma and normal pancreatic tissues were evaluated using the Human Protein Atlas. Finally, functional analyses were performed in pancreatic ductal adenocarcinoma (PDAC). Results: *LGALS3* deletion across multiple cancer cell lines led to transcriptomic changes involving mitotic progression, stress responses, and axonal guidance pathways. High *LGALS3* expression was significantly associated with worse overall survival in lower-grade glioma, PDAC, uveal melanoma, and kidney renal papillary cell carcinoma. *LGALS3* knockout in YAPC cells recapitulated the pan-cancer findings, linking *LGALS3* to cell morphogenesis and proliferation. Conclusions: These findings identify Galectin-3 as a key regulator of oncogenic programs and a potential prognostic biomarker in PDAC and other malignancies, with implications for therapeutic targeting.

## 1. Introduction

Galectins constitute a family of evolutionarily conserved β-galactoside-binding proteins characterized by the presence of carbohydrate recognition domains (CRDs), which mediate interactions with glycoproteins and glycolipids in a calcium-independent manner [1,2]. Among them, Galectin-3 (Gal-3), encoded by the LGALS3 gene, is unique in structure and function, possessing a chimeric architecture that enables it to oligomerize and mediate crosslinking of cell surface glycoconjugates. Gal-3 is localized in the nucleus, cytoplasm, and extracellular space, and it plays a central role in a diverse array of biological processes, including cell adhesion, proliferation, apoptosis, immune regulation, angiogenesis, and inflammation [3,4].

In the context of cancer, Gal-3 has emerged as a multifunctional oncoprotein. Elevated LGALS3 expression has been reported in a variety of malignancies, including glioblastoma, breast cancer, melanoma, colorectal cancer, hepatocellular carcinoma, and pancreatic ductal adenocarcinoma [5,6,7]. Functionally, Gal-3 enhances tumor progression through several mechanisms, including inhibition of apoptosis, promotion of epithelial–mesenchymal transition (EMT), stimulation of angiogenesis, and modulation of immune responses [2,8]. Moreover, Gal-3 facilitates cancer cell invasion and metastasis by remodeling the extracellular matrix (ECM) and stabilizing receptor tyrosine kinases (RTKs) on the cell surface, thereby sustaining oncogenic signaling [8].

Pancreatic ductal adenocarcinoma (PDAC) is among the deadliest forms of cancer, with a 5-year survival rate of less than 10% [9]. The dismal prognosis is attributed to late diagnosis, aggressive tumor biology, extensive stromal fibrosis, and profound resistance to conventional therapies. Emerging evidence suggests that Gal-3 plays a critical role in PDAC pathogenesis. Gal-3 is highly expressed in both cancer cells and the tumor-associated stroma, where it contributes to tumor cell survival, immune evasion, and resistance to chemotherapy. In vitro studies have shown that Gal-3 binds directly to activated Ras proteins, prolonging their GTP-bound state and enhancing downstream MAPK and PI3K-AKT signaling cascades [6]. These findings are particularly relevant to PDAC, in which activating mutations in KRAS occur in over 90% of tumors and serve as the principal oncogenic driver.

Despite the increasing interest in Gal-3 as both a therapeutic target and prognostic biomarker, the full extent of its functional roles in cancer—particularly at the transcriptomic level—remains incompletely characterized. Previous studies have primarily focused on individual signaling pathways or cellular processes regulated by Gal-3, often in specific cancer contexts. However, a systematic, pan-cancer analysis of the molecular and clinical consequences of LGALS3 dysregulation is lacking. Moreover, the precise gene networks and biological programs downstream of Gal-3 have not been comprehensively defined using genome-wide approaches. This knowledge gap limits our understanding of how Gal-3 integrates into broader oncogenic signaling networks and constrains the development of targeted therapies.

Importantly, Gal-3 also plays a pivotal role in shaping the tumor immune microenvironment (TME). It has been implicated in the recruitment and functional suppression of tumor-infiltrating lymphocytes (TILs), including CD8^+^ T cells, natural killer (NK) cells, and dendritic cells [10]. Through interactions with glycosylated immune checkpoints, such as LAG-3 and TIM-3, Gal-3 inhibits T-cell activation and fosters immune exhaustion [11]. In parallel, Gal-3 promotes the expansion of myeloid-derived suppressor cells (MDSCs) and M2-polarized macrophages, contributing to an immune-suppressive TME that supports tumor growth and resistance to immunotherapy [12]. These immunomodulatory functions position Gal-3 at the crossroads of oncogenesis and immune escape, making it an attractive target for combination therapies aimed at reinvigorating anti-tumor immunity.

Given its multifaceted role in cancer biology, Gal-3 has emerged as a therapeutic target. Several Gal-3 inhibitors—including belapectin (GR-MD-02), GB1211, and TD139—are currently in preclinical or early clinical development for cancer and fibrotic diseases [13]. However, rational deployment of Gal-3-targeted therapies in the clinic will require a more detailed understanding of its downstream effects and the molecular context in which it exerts oncogenic functions.

In this study, we sought to comprehensively investigate the transcriptomic and clinical significance of LGALS3 expression in cancer, with a particular focus on pancreatic ductal adenocarcinoma (PDAC). First, we aimed to define the transcriptional programs regulated by LGALS3 through CRISPR-mediated gene deletion and subsequent gene ontology analysis. Second, we evaluated the prognostic relevance of LGALS3 across diverse cancer types using pan-cancer datasets from The Cancer Genome Atlas (TCGA), examining its association with overall survival in multivariable models adjusted for tumor stage and purity. Third, we investigated the expression profile of LGALS3 in pancreatic ductal adenocarcinoma (PDAC), integrating transcriptomic and immunohistochemical data to assess both RNA and protein levels. Finally, we identified LGALS3-associated gene co-expression networks in PDAC and performed pathway enrichment analyses to uncover the biological processes and signaling pathways linked to Gal-3 activity in this tumor type.

Collectively, this integrative analysis aims to provide a systems-level understanding of Gal-3′s role in tumorigenesis, thereby providing the rationale for the development of Gal-3-targeted intervention in PDAC and other cancers.

## 2. Materials and Methods

### 2.1. Consensus Signature and MCODE Analysis from SIGCOM LINCS

To investigate the functional impact of LGALS3 deletion across cancer cell lines, we used the precomputed consensus transcriptomic signature of LGALS3 CRISPR knockout provided by the SIGCOM LINCS platform (https://maayanlab.cloud/sigcom-lincs/) (accessed on 3 June 2025) [14]. This consensus signature was derived by multiple independent CRISPR perturbation experiments and represents the core set of transcriptional changes associated with LGALS3 loss. Pathway enrichment and network module analysis were performed using the Metascape web tool (https://metascape.org) (accessed on 3 June 2025) [15], which enables integrative bioinformatic analysis by using the MCODE (Molecular Complex Detection) algorithm, that allows to identify densely connected network modules. Functional annotation was based on Gene Ontology (GO), KEGG, and Reactome databases. Significantly enriched clusters were defined by an enrichment factor > 1.5, a minimum gene count of 3, and a *p*-value < 0.01.

### 2.2. Pan-Cancer Survival Analysis

The prognostic value of LGALS3 expression across multiple cancer types was systematically evaluated using the TIMER2.0 webserver (http://timer.cistrome.org/) (accessed on 3 June 2025) [16], a comprehensive resource for immune-related and survival analyses based on The Cancer Genome Atlas (TCGA) data. TIMER2.0 incorporates multivariate Cox proportional hazards modeling to assess gene expression–survival associations while adjusting for key confounding factors, including tumor stage and tumor purity. For this analysis, pan-cancer datasets were queried to assess 60-month overall survival (OS) outcomes. Patients were stratified into high and low LGALS3 expression groups using the 75th and 25th percentile cut-offs, respectively. Hazard ratios (HRs), 95% confidence intervals (CIs), and *p*-values were calculated, with statistical significance defined as *p* < 0.05. Kaplan–Meier survival curves were generated for tumor types in which LGALS3 expression demonstrated a statistically significant association with overall survival, providing visual representation of the prognostic impact of LGALS3 expression in those cancers.

### 2.3. Expression Analysis of LGALS3 in Pancreatic Cancer

To evaluate LGALS3 expression specifically in pancreatic ductal adenocarcinoma (PDAC) and normal tissue, transcript-level data were retrieved from the UCSC Xena browser (https://xenabrowser.net/) (accessed on 3 June 2025). Galectin-3 protein expression was assessed using immunohistochemistry (IHC) data available in the HPA, generated with the HPA003162 antibody and available from the Human Protein Atlas (HPA, https://www.proteinatlas.org) (accessed on 3 June 2025). IHC images and associated metadata were reviewed to determine staining intensity (categorized as weak, moderate, or strong) and the proportion of positively stained cells. Comparative evaluation was performed across normal pancreatic tissues and PDAC samples to characterize potential differences in LGALS3 protein expression patterns between physiological and malignant states. Differences in LGALS3 expression between primary and metastatic PDAC tumors were evaluated by interrogating the GSE205154 dataset. Trimmed mean of M-values (TMM)–normalized data were used for the differential expression analysis to ensure appropriate adjustment for library size and compositional biases. The dataset included 207 primary tumors and 70 metastatic samples.

### 2.4. Co-Expression and Functional Correlation in PDAC

To construct LGALS3-centered co-expression networks in pancreatic ductal adenocarcinoma (PDAC), a genome-wide correlation analysis was conducted using TCGA-PAAD transcriptomic data accessed via the cBioPortal platform (https://www.cbioportal.org) (accessed on 3 June 2025). Spearman correlation coefficients (ρ) were calculated between LGALS3 and all other protein-coding genes. Genes exhibiting a correlation coefficient ρ > 0.5 and false discovery rate (FDR) < 0.05 were considered significantly co-expressed with LGALS3. The resulting gene set was then subjected to functional enrichment analysis using the Metascape web tool (https://metascape.org) (accessed on 3 June 2025) to identify overrepresented biological processes, molecular functions, and canonical pathways. Enrichment results were filtered using the following criteria: enrichment factor > 1.5, minimum gene count of 3, and *p* < 0.01, to ensure both biological relevance and statistical robustness.

### 2.5. Consensus Signature Derivation for YAPC Cell Line

To derive cell line-specific consensus signature, we retrieved two independent CRISPR-mediated LGALS3 knockout gene expression profiles for the YAPC pancreatic cancer cell line at 96 h post-perturbation from the SIGCOM LINCS database (https://maayanlab.cloud/sigcom-lincs) (accessed on 3 June 2025). To derive a robust transcriptional response, the two perturbation signatures were meta-analyzed using the RankProduct method, generating a consensus LGALS3 knockout signature specific to the YAPC cell line. Differentially expressed genes (DEGs) were identified through 10,000 permutations to compute empirical *p*-values, with statistical significance set at *p* < 0.05. Functional enrichment analysis of DEGs was performed using Metascape (accessed on 3 June 2025), separately analyzing upregulated and downregulated gene sets to uncover distinct biological processes associated with LGALS3 loss. Enrichment results were filtered using stringent thresholds: enrichment factor > 1.5, minimum gene count ≥ 3, and *p* < 0.01, ensuring the biological relevance and robustness of the findings.

### 2.6. Statistical Analysis

All statistical analyses were performed using Prism (version 9.0) or via web-based interfaces. The RankProduct method used 10,000 permutations to determine significance. For survival analysis, TIMER2.0 internally uses Cox regression models and the log-rank test for Kaplan–Meier analysis. Significance was defined as a *p*-value < 0.05 unless otherwise stated.

## 3. Results

### 3.1. Transcriptomic Impact of LGALS3 Deletion in Cancer Cell Lines

**Figure 1:**
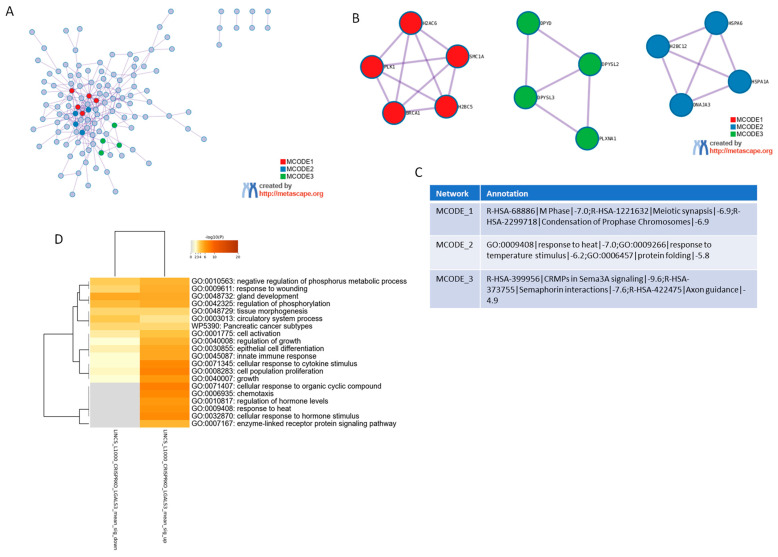
Functional enrichment analysis of the consensus gene signature following LGALS3 CRISPR deletion. (**A**) Protein–Protein Interaction network of genes consistently deregulated across multiple cancer cell lines upon LGALS3 knockout, conducted using the Metascape platform (accessed on 3 June 2025). Each MCODE network is assigned a unique color. (**B**) Network visualization of MCODE clusters, representing densely connected functional modules. (**C**) Summary table of major functional clusters enriched in the consensus gene signature, highlighting representative biological processes and pathways associated with LGALS3 loss. (**D**) Gene Ontology (GO) and pathway enrichment analysis of the consensus signature derived from CRISPR/Cas9-mediated knockout of LGALS3 is presented as heatmap. The heatmap cells are colored by their *p*-values and significant terms are hierarchically clustered based on their statistical similarities.

To investigate the molecular effects associated with LGALS3 loss, we analyzed a consensus gene expression profile generated from CRISPR-Cas9-mediated deletion of LGALS3 across multiple cancer cell lines. The data were obtained from the SigCom LINCS repository (accessed on 3 June 2025), which compiles transcriptomic signatures following genetic perturbations. The gene list representing LGALS3 knockout was subjected to gene ontology enrichment analysis using Metascape (accessed on 3 June 2025) (Figure 1A). The resulting network analysis revealed three major molecular complexes identified through the MCODE algorithm, each representing a distinct group of functionally related genes (Figure 1B). The first module, MCODE_1, was strongly enriched in pathways associated with cell cycle control. Specifically, this cluster included genes involved in “M phase” (Reactome: R-HSA-68886), “meiotic synapsis” (R-HSA-1221632), and “condensation of prophase chromosomes” (R-HSA-2299718). These terms were consistently enriched with high significance and involved core regulators of mitotic spindle assembly and chromosomal dynamics. The second module, MCODE_2, was enriched in pathways linked to cellular stress responses, including “response to heat” (GO:0009408), “response to temperature stimulus” (GO:0009266), and “protein folding” (GO:0006457). The third module, MCODE_3, consisted of genes participating in signaling pathways related to neural and axonal development. This cluster included “CRMPs in Sema3A signaling” (R-HSA-399956), “semaphorin interactions” (R-HSA-373755), and “axon guidance” (R-HSA-422475). These categories grouped genes involved in cytoskeletal organization and signal transduction related to directional cell movement and intercellular signaling (Figure 1C). Together, the modular analysis based on LGALS3 deletion revealed discrete gene networks involved in cell division, stress adaptation, and signaling processes that are shared among multiple cancer cell models. Gene Ontology (GO) and pathway enrichment analysis of the consensus signature derived from CRISPR/Cas9-mediated knockout of LGALS3 also revealed distinct functional programs (Figure 1D), including processes such as cellular response to cytokine stimulus (GO:0071345), cell population proliferation (GO:0008283), innate immune response (GO:0045087), chemotaxis (GO:0006935), and cellular response to hormone stimulus (GO:0032870), which displayed the highest significance scores. In addition, enrichment of terms related to epithelial cell differentiation (GO:0030855) and tissue morphogenesis points to a shift in differentiation and remodeling programs. Importantly, a cancer-relevant pathway, Pancreatic cancer subtypes (WP5390), also emerged as significantly modulated (Figure 1D).

### 3.2. Prognostic Value of LGALS3 Expression in Human Cancers

**Figure 2:**
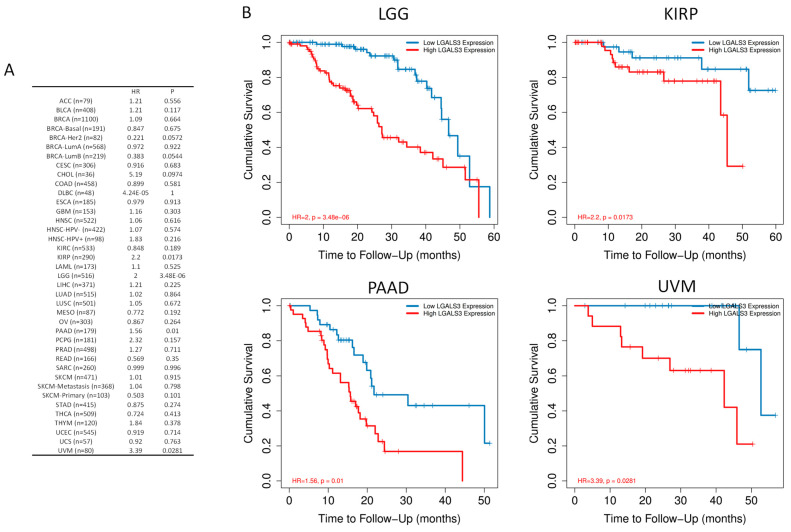
Prognostic significance of LGALS3 expression across human cancers. (**A**) Hazard ratios (HRs) and corresponding *p*-values for overall survival (OS) at 60 month follow-up across TCGA tumor types, stratified by LGALS3 expression and adjusted for tumor stage and purity using multivariate Cox regression. (**B**) Kaplan–Meier survival curves illustrating significantly reduced OS in patients with high LGALS3 expression for selected cancer types where *p* < 0.05.

To assess the clinical associations of LGALS3 expression, an exploratory pan-cancer survival analysis was performed using RNA-seq data and matched clinical annotations from The Cancer Genome Atlas (TCGA), implemented in TIMER2.0 web-based utility. The analysis included all major tumor types and was adjusted for potential confounding factors such as tumor stage and tumor purity. The results identified four cancer types in which high LGALS3 expression levels were significantly associated with decreased overall survival (Figure 2A). In lower-grade glioma (LGG), high LGALS3 expression corresponded to a hazard ratio (HR) of 2.0 with a *p*-value of 3.48 × 10^−6^. In pancreatic adenocarcinoma (PAAD), elevated expression yielded an HR of 1.56 and a *p*-value of 0.01. In uveal melanoma (UVM), a stronger effect was observed with an HR of 3.39 and a *p*-value of 0.0281. In kidney renal papillary cell carcinoma (KIRP), the HR was 2.2, with a *p*-value of 0.0173 (Figure 2B).

### 3.3. Gal-3 Expression and Function in Pancreatic Ductal Adenocarcinoma (PDAC)

**Figure 3:**
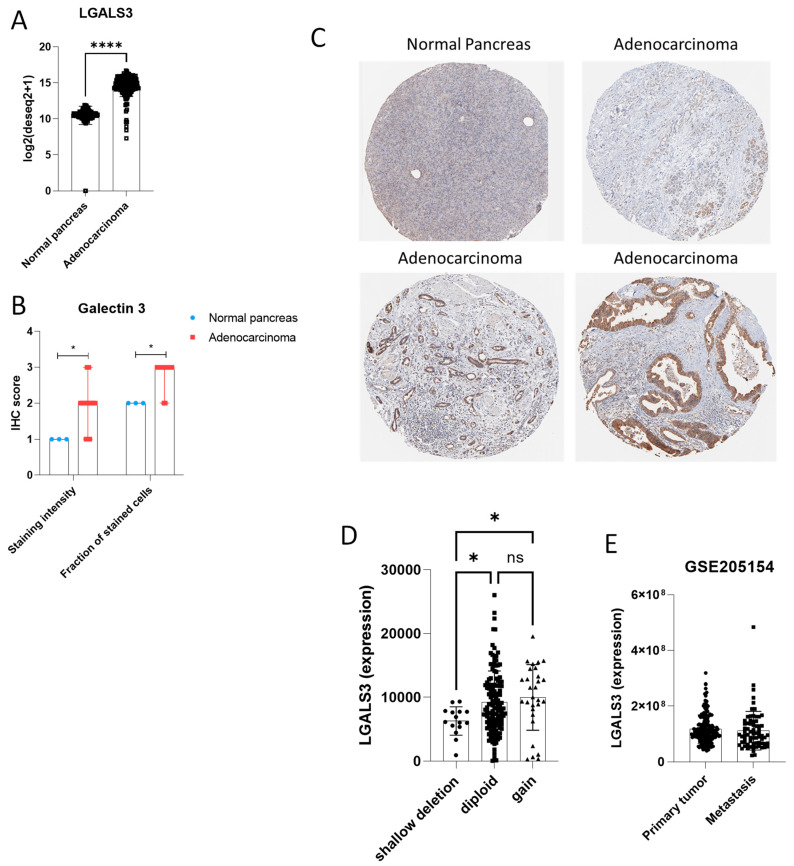
LGALS3 expression in pancreatic ductal adenocarcinoma (PDAC). (**A**) RNA expression levels of LGALS3 in PDAC tissues from the TCGA and GTEx cohort. (**B**) Immunohistochemistry staining for Galectin-3 in normal pancreas and PDAC samples using antibody HPA003162. (**C**) Representative immunohistochemistry images for Galectin-3 in normal pancreas and PDAC samples using antibody HPA003162. (**D**) LGALS3 expression in PDAC tissues from TCGA, grouped by copy number status (shallow deletion, diploid, gain). (**E**) LGALS3 expression levels in primary and metastatic PDAC tumors from the GSE205154 dataset. * *p* < 0.05; **** *p* < 0.0001; ns = not significant.

Based on the prognostic associations observed in pancreatic cancer, we next examined LGALS3 expression in pancreatic ductal adenocarcinoma (PDAC) at both transcriptomic and proteomic level (Figure 3A,B). Transcript levels of LGALS3 were significantly higher in pancreatic adenocarcinoma samples as compared to normal pancreas sample (Figure 3A), although a fraction of samples showed overlapping LGALS3 levels with the normal tissue counterpart. Accordingly, at the protein level, immunohistochemistry data from the Human Protein Atlas, using antibody HPA003162, confirmed increased Galectin-3 staining in PDAC specimens (Figure 3B,C). No recurrent mutations or structural variants were identified in LGALS3. CNAs were observed in a subset of cases: 15 shallow deletions, 29 gains, and the majority (132 samples) were diploid without alteration (Figure 3D). Expression analysis showed that shallow deletions were associated with significantly reduced LGALS3 expression compared to diploid (*p* = 0.0238, q = 0.0125) and gain (*p* = 0.0158, q = 0.0125) groups. No significant difference was observed between diploid and gain groups (Figure 3D). Finally, we analyzed the LGALS3 expression in 70 metastatic and 207 primary PDAC samples, from the GSE205154 dataset. Our differential analysis revealed no statistically significant difference in LGALS3 expression between primary and metastatic tumors (Figure 3E). In addition, we analyzed the relationship between LGALS3 and LGALS3BP in TCGA-PAAD tumors. Our results revealed a moderate but statistically significant positive correlation (Spearman ρ = 0.38, *p* = 2.32 × 10^−7^) (Supplementary Figure S2), indicating that these genes may be co-regulated in pancreatic cancer and potentially linked through a feedback mechanism. Interestingly, in contrast, the YAPC LGALS3 knockout signature did not show significant modulation of LGALS3BP, suggesting that the regulation observed in patient tumors may not be directly mediated by LGALS3 loss, or that additional tumor microenvironmental or systemic factors are required for this regulation.

**Figure 4:**
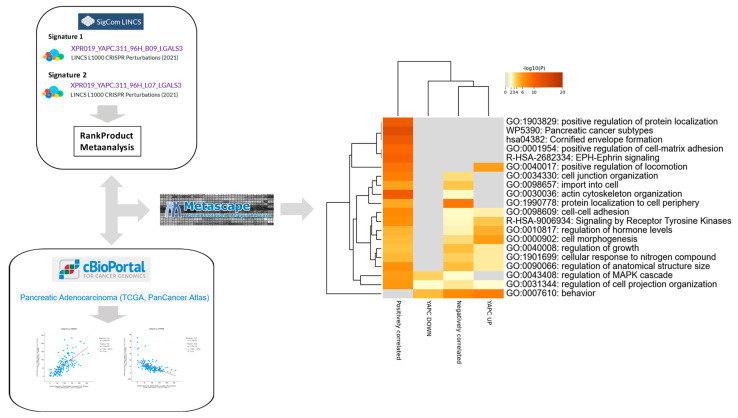
Convergent pathway enrichment between YAPC LGALS3 knockout transcriptomes and LGALS3-correlated genes in TCGA-PAAD. Hierarchical clustering of Gene Ontology (GO) and pathway enrichment scores for differentially expressed genes from YAPC pancreatic cancer cells following CRISPR/Cas9-mediated knockout of LGALS3, together with genes significantly correlated with LGALS3 expression in TCGA-PAAD. The heatmap cells are colored by their *p*-values and significant terms are hierarchically clustered based on their statistical similarities.

To integrate experimental and clinical evidence, we next compared the functional pathways emerging from LGALS3 knockout in the YAPC pancreatic cancer cell line with those identified through co-expression analysis of LGALS3 in TCGA-PAAD tumors, aiming to define convergent biological programs associated with galectin-3 in pancreatic cancer (Figure 4). Hierarchical clustering of enrichment profiles showed that YAPC downregulated genes clustered most closely with positively correlated genes in TCGA PDAC, whereas YAPC upregulated genes clustered with negatively correlated TCGA genes, indicating substantial overlap between experimental and clinical datasets.

Both sets of data displayed strong enrichment for cell–cell adhesion processes, including cell–cell adhesion (GO:0098609, −log*p* 2.59–7.17) and cell junction organization (GO:0034330, −log*p* 3.28–8.05). Signaling pathways were also consistently represented, in particular Receptor Tyrosine Kinase signaling (R-HSA-9006934, −log*p* 3.92–6.86) and regulation of MAPK cascade (GO:0043408, −log*p* 3.60–5.92). Categories related to growth control were enriched across datasets, including regulation of growth (GO:0040008, −log*p* 2.78–4.23), regulation of anatomical structure size (GO:0090066, −log*p* 2.78–6.51), and cell morphogenesis (GO:0000902, −log*p* 4.48–5.78). Structural and motility-related categories were also convergent, including cell projection organization (GO:0031344, −log*p* 2.04–6.11) and actin cytoskeleton organization (GO:0030036, −log*p* 2.23–11.17). Finally, the category behavior (GO:0007610) was significantly enriched in both datasets (–log*p* 4.39–7.89), further indicating overlap in transcriptional regulation (Figure 4). Notably, several Gene Ontology categories were consistently enriched not only in YAPC cells and TCGA-PAAD correlated genes but also in the consensus pancancer LGALS3-CRISPRKO signature (Supplementary Figure S1). Shared pathways included cell–cell adhesion (GO:0098609), regulation of the MAPK cascade (GO:0043408), regulation of growth (GO:0040008), cell projection organization (GO:0031344), and behavior (GO:0007610) (Supplementary Figure S1).

## 4. Discussion

Galectin-3 (Gal-3), encoded by the LGALS3 gene, is a β-galactoside-binding lectin that has been implicated in multiple cellular processes, including proliferation, apoptosis, adhesion, migration, and immune modulation [17]. Aberrant expression of LGALS3 has been described in a variety of cancers, such as glioblastoma, colorectal cancer, hepatocellular carcinoma, thyroid carcinoma, and pancreatic ductal adenocarcinoma (PDAC) [18]. Gal-3 is unique among galectins because of its chimeric structure, which allows oligomerization and crosslinking of glycoproteins on the cell surface and in the extracellular matrix. This structural feature enables Gal-3 to function as a scaffolding molecule, coordinating multiple signaling pathways and mediating interactions between tumor cells and the surrounding microenvironment [19].

In this study, we evaluated LGALS3 expression and functional relevance in PDAC through a combined experimental and computational approach. We have analyzed CRISPR-Cas9-mediated knockout of LGALS3 in YAPC cells and evaluated the resulting transcriptomic changes. In parallel, we assessed LGALS3 expression patterns in TCGA PDAC datasets and integrated these findings with survival data. This dual approach allowed us to define conserved Gal-3–dependent pathways in PDAC and evaluate their clinical relevance.

Our analyses revealed that LGALS3 is consistently upregulated in PDAC tumors compared to normal pancreatic tissue, both at the RNA and protein levels. Immunohistochemical data from the Human Protein Atlas corroborated this observation, showing moderate to strong Gal-3 staining in tumor cells. Importantly, when comparing primary and metastatic PDAC samples within the TCGA cohort (207 primary, 70 metastatic), we found no significant differences in LGALS3 expression. This indicates that Gal-3 overexpression is likely an early event in PDAC tumorigenesis and is maintained during metastatic progression, rather than being selectively enriched in metastatic lesions. Such a pattern suggests that LGALS3 may contribute to tumor maintenance and general aggressiveness, rather than specifically promoting metastasis.

Functional enrichment analyses of LGALS3 knockout cells revealed significant perturbations in pathways related to cell cycle regulation, protein homeostasis, and cytoskeletal organization. Specifically, genes involved in mitotic progression, chromosome condensation, and proteostasis were downregulated upon LGALS3 deletion. These findings are consistent with previous reports that Gal-3 supports the G1/S and G2/M transitions, maintains mitotic fidelity, and enhances tumor cell survival under stress conditions [19,20,21]. In addition, genes associated with cytoskeletal remodeling, axon guidance, and semaphorin signaling were altered, reflecting Gal-3′s role in regulating cell adhesion, motility, and invasion [22,23]. Integration of these data with TCGA PDAC transcriptomes highlighted a conserved network of Gal-3–associated processes, including cell–cell adhesion, receptor tyrosine kinase signaling, MAPK regulation, and cytoskeletal organization. This overlap highlights the biological relevance of Gal-3 in clinical PDAC tumors and suggests that it orchestrates multiple oncogenic programs to sustain tumor growth and adaptability [24,25,26,27,28].

Our exploratory survival analysis further indicated that elevated LGALS3 expression correlates with poorer overall survival in PDAC and several other tumor types, including low-grade glioma (LGG), uveal melanoma (UVM), and kidney renal papillary cell carcinoma (KIRP). Although TCGA-based survival analysis cannot establish causality, these findings are consistent with functional studies demonstrating that Gal-3 promotes tumor cell proliferation, resistance to apoptosis, and evasion of immune surveillance. In PDAC, Gal-3 directly binds and stabilizes active GTP-bound Ras proteins, sustaining downstream MAPK/ERK and PI3K/AKT signaling, which is particularly relevant given the high prevalence of KRAS mutations in pancreatic cancer. By amplifying Ras signaling, Gal-3 likely contributes to the aggressive growth and therapy resistance observed in PDAC [6].

In addition to tumor-intrinsic effects, Gal-3 exerts profound influence on the tumor microenvironment (TME). It is expressed by tumor cells, stromal fibroblasts, and tumor-associated macrophages, and can modulate immune cell recruitment and polarization. Gal-3 interactions with glycoproteins on immune cells facilitate expansion of myeloid-derived suppressor cells and M2-like macrophage polarization, suppressing cytotoxic T-cell responses and promoting immune evasion. Gal-3 also binds checkpoint receptors such as LAG3 on T cells, further dampening anti-tumor immunity. These findings suggest that Gal-3 functions as a multi-faceted immunoregulatory molecule in PDAC, supporting tumor progression both directly and indirectly via TME modulation [29,30].

Interestingly, despite its established roles in invasion and metastasis in other tumor models [31,32,33,34,35], our data indicate that LGALS3 expression is similar in primary and metastatic PDAC tumors. This suggests that while Gal-3 contributes to aggressive tumor biology and immune evasion, additional factors may govern metastatic dissemination in PDAC, such as stromal remodeling, epithelial-to-mesenchymal transition, or interactions with the extracellular matrix. In other words, Gal-3 may set the stage for tumor aggressiveness without being the primary driver of metastasis.

From a therapeutic perspective, the central role of Gal-3 in PDAC biology highlights it as a promising target. Several Gal-3 inhibitors, including belapectin (GR-MD-02) and GB1211, are in preclinical or early-phase clinical development, with potential applications in combination with immune checkpoint blockade [36,37,38]. Given the immunosuppressive nature of PDAC, combining Gal-3 inhibition with anti-PD-1 or anti-CTLA-4 therapies could enhance anti-tumor immunity. Furthermore, LGALS3 expression could serve as a predictive biomarker for identifying patients likely to benefit from such interventions.

Despite these insights, several limitations should be acknowledged. First, the CRISPR-Cas9 knockout studies were performed in established PDAC cell lines, which, although valuable for mechanistic exploration, cannot fully replicate the complex architecture of human tumors, including stromal and immune interactions. Second, TCGA data represent bulk RNA-seq measurements, limiting cell-type resolution and potentially confounding interpretation due to heterogeneity in stromal content, immune infiltration, and mutational background. Single-cell RNA-sequencing or spatial transcriptomics could provide more precise information on cell-specific LGALS3 expression. Third, while our study demonstrates robust associations between LGALS3 expression, tumor biology, and patient outcomes, causality remains to be fully established. Functional validation in orthotopic or genetically engineered mouse models, patient-derived xenografts, or organoid co-culture systems will be essential to confirm the role of Gal-3 in vivo.

In conclusion, our study provides comprehensive evidence that LGALS3 functions as a multifunctional oncogene in PDAC, integrating cell cycle control, cytoskeletal organization, stress response, and immunomodulation. LGALS3 overexpression is a consistent feature in both primary and metastatic PDAC, supporting its role in maintaining tumor aggressiveness rather than driving metastasis. These findings establish a framework for future therapeutic strategies targeting Gal-3, either alone or in combination with immunotherapy, and underscore its potential as a biomarker of tumor aggressiveness. Overall, this work provides a focused, mechanistic, and clinically relevant perspective on LGALS3 in PDAC, while highlighting directions for further validation and translational application.

## Data Availability

All data presented in the paper are publicly available from the indicated sources.

## Supplementary Materials

The following supporting information can be downloaded at: https://www.mdpi.com/article/10.3390/genes16101170/s1, Figure S1: Convergent pathway enrichment between the SigCom LINCS pan-cancer LGALS3 knockout signature, YAPC LGALS3 knockout transcriptomes, and LGALS3-correlated genes in TCGA-PAAD; Figure S2: Correlation between LGALS3 and LGALS3BP expression in TCGA-PAAD tumors.
