# Supplementary figures and images for: Transcriptomic and Clinical Profiling Reveals LGALS3 as a Prognostic Oncogene in Pancreatic Cancer
Source: Genes (Basel). 2025 Oct 3;16(10):1170. doi: 10.3390/genes16101170 (PMC12562417; doi:10.3390/genes16101170)

## Slide 1
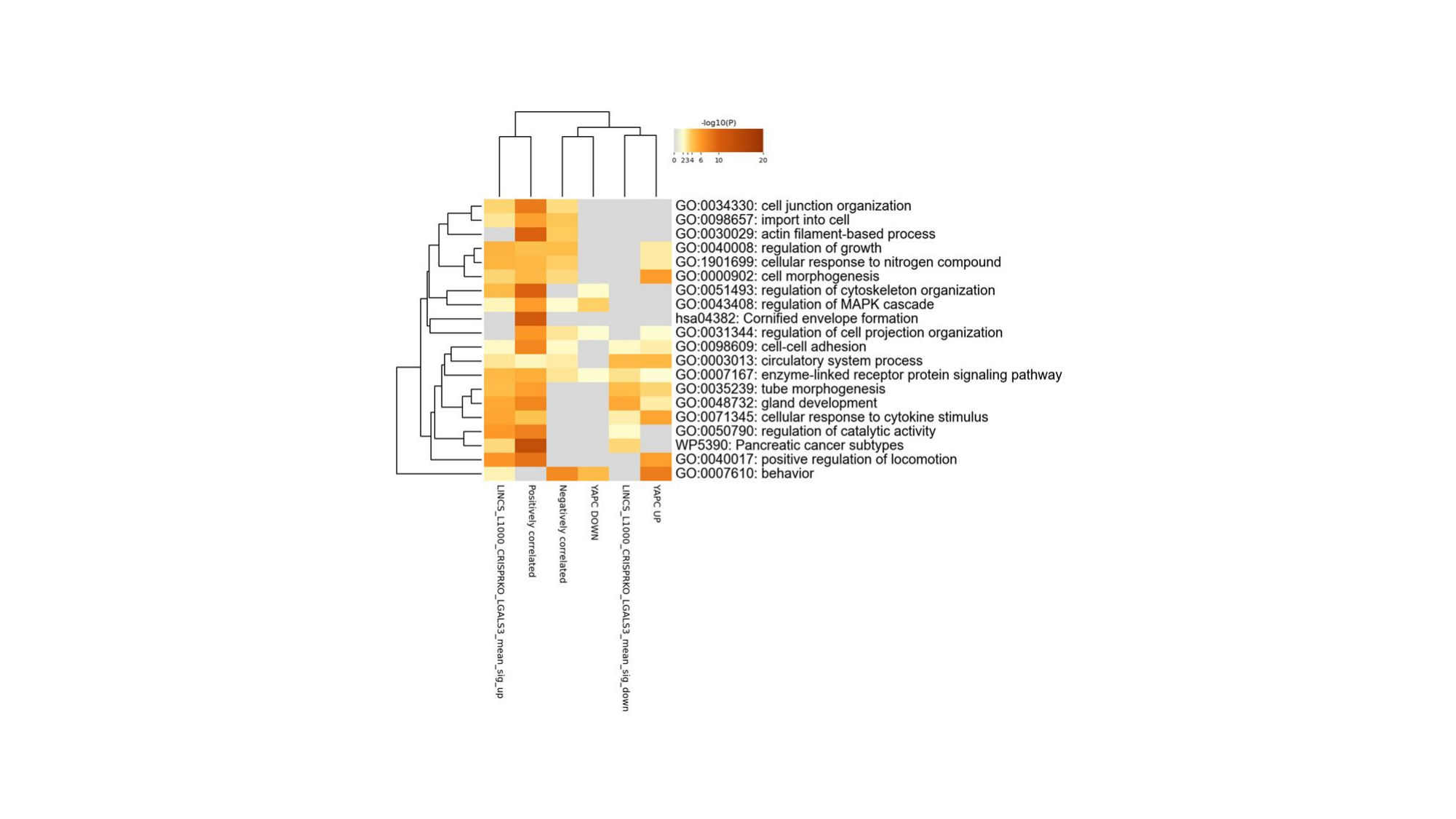

## Slide 2
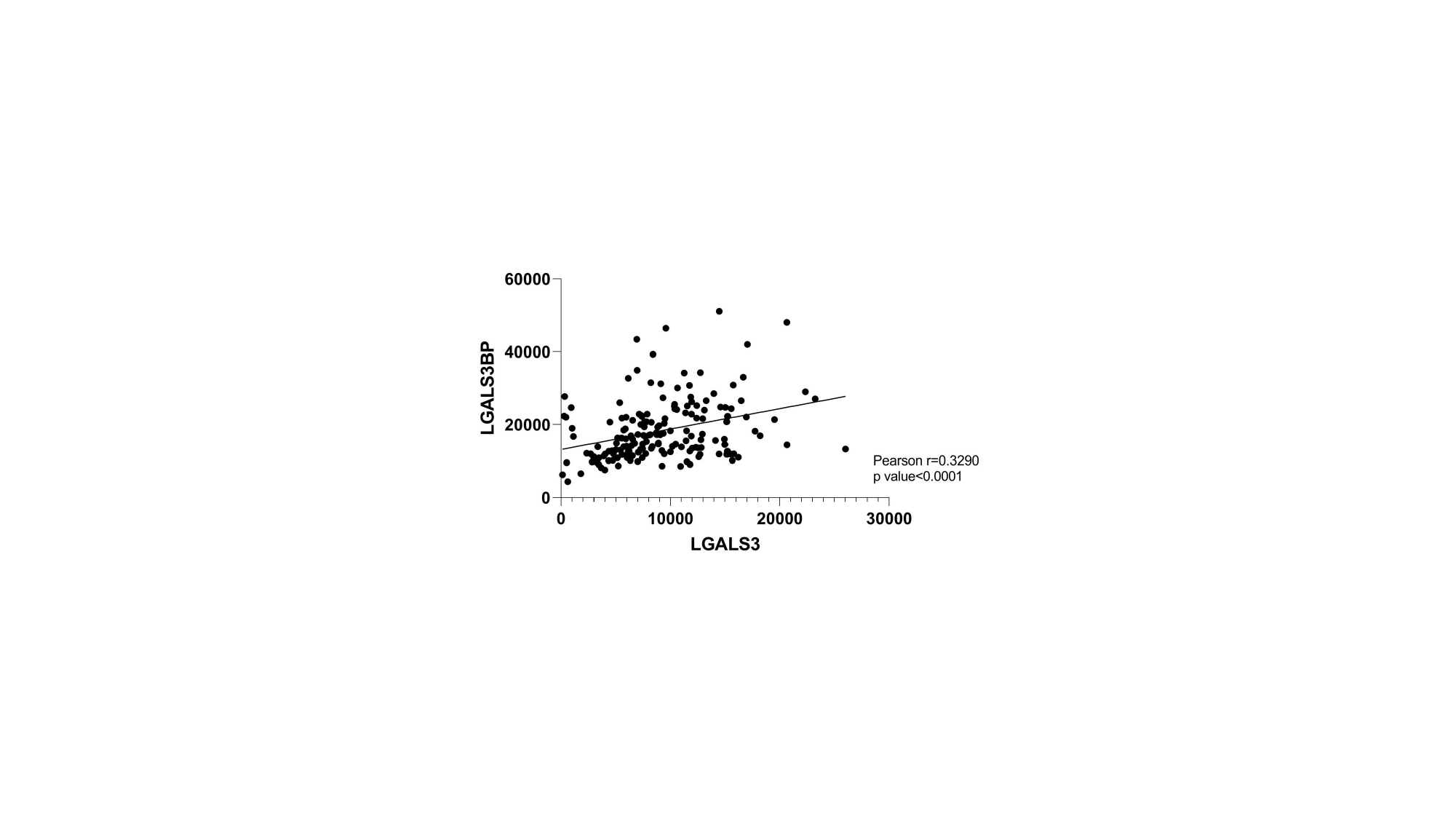

Supplement: Supplementary file 1 [file genes-16-01170-s001.zip › genes-3865368-supplementary.pptx]
